# Supplementary material for: Future sea ice weakening amplifies wind-driven trends in surface stress and Arctic Ocean spin-up
Source: Nat Commun. 2024 Aug 12;15:6889. doi: 10.1038/s41467-024-50874-0 (PMC11319342; doi:10.1038/s41467-024-50874-0)
Supplement: Supplementary file 1 — Supplementary Information [file 41467_2024_50874_MOESM1_ESM.pdf]

# Supplementary Information to "Future sea ice weakening amplifies wind-driven trends in surface stress and Arctic Ocean spin-up"

Morven Muilwijk[1*], Tore Hattermann[1,2], Torge Martin[3], Mats A. Granskog[1]

[1*]Norwegian Polar Institute, Fram Centre, Tromsø, Norway.
[2]Complex Systems Group, Department of Mathematics and Statistics, UiT - The Arctic University of Norway, Tromsø, Norway.
[3]GEOMAR Helmholtz Centre for Ocean Research, Kiel, Germany.

*Corresponding author(s). E-mail(s): morven.muilwijk@npolar.no;

## 1 Supplementary Figures

This file includes:

1. Supplementary Fig. 1
2. Supplementary Fig. 2
3. Supplementary Fig. 3
4. Supplementary Fig. 4

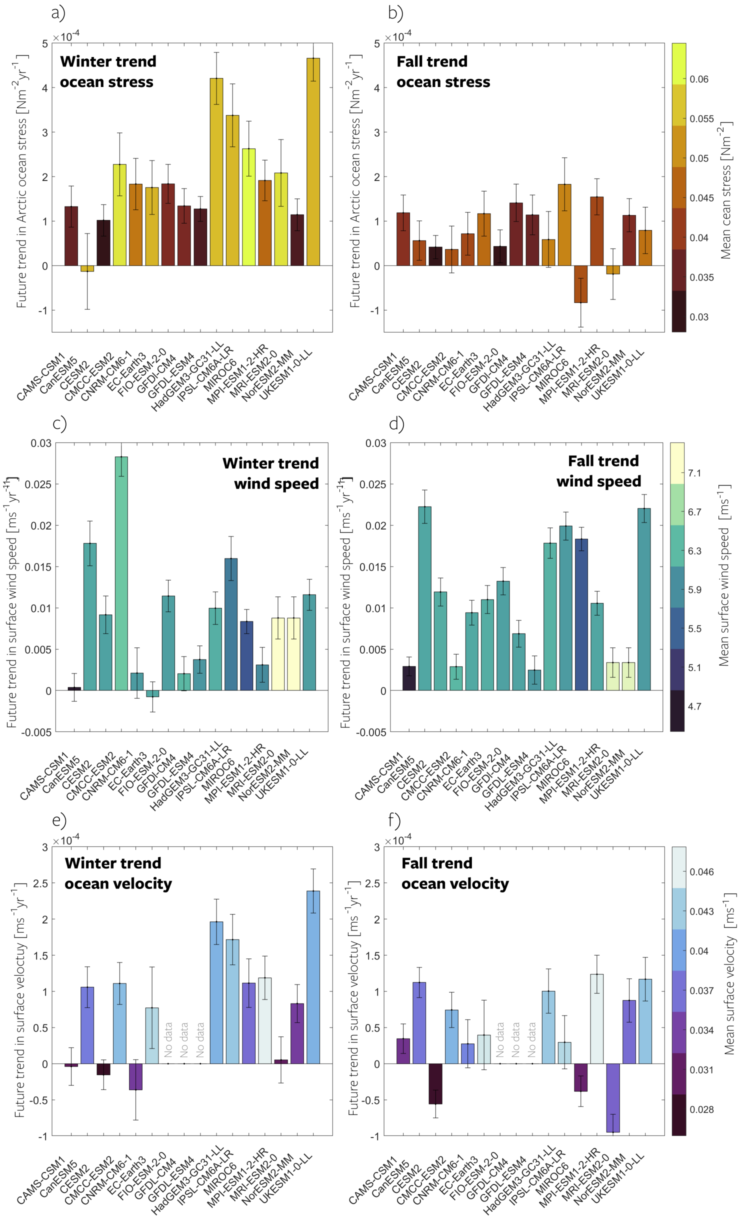


**Supplementary Fig. 1** Individual model trends of future stress, surface wind speed, and stress. Simulated trends in ocean stress (a, b), surface wind speed (c, d), and ocean surface velocity (e, f) for each of the CMIP6 models listed in Table S1 over the period 2015-2100. The left column displays the trends during winter (January-March), while the right column shows the trends for fall (September-November). Color bars represent the mean state for each model, and error bars indicate the standard error at a 95% confidence level.

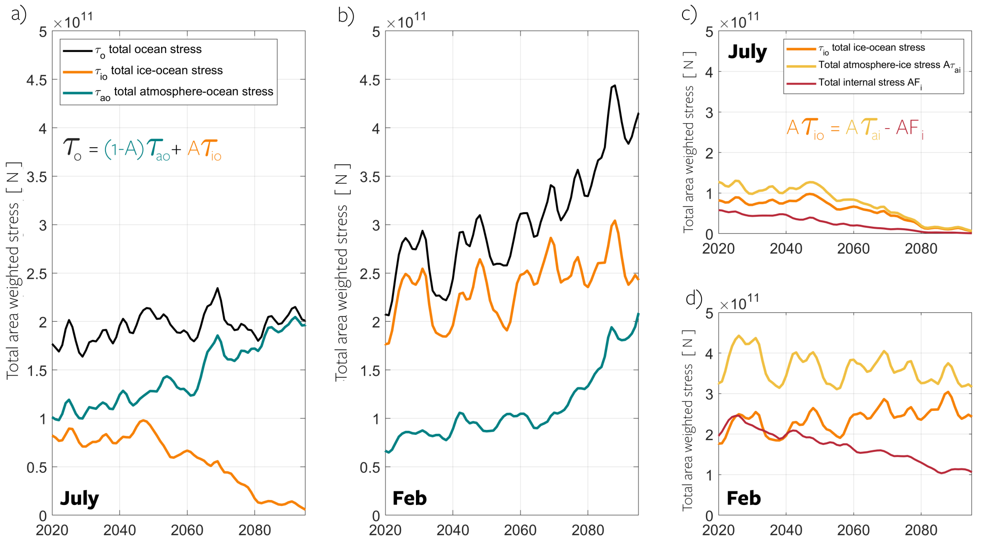


**Supplementary Fig. 2** Time series of decomposed stress terms in NorESM2-MM. (a) Time series of total area-integrated ocean surface stress (black line) in the NorESM2-MM model (Methods), showcasing the relative contributions of total ice-ocean stress (orange line) and atmosphere-ocean stress (teal line) for a summer month (July). (b) Same as for (a), but for a winter month (February). c) Time series of total area-integrated ice-ocean stress (orange line) in the NorESM2-MM model decomposed into total atmosphere-ice stress (yellow line) and internal stress (red line) for a summer month (July). (d) Same as (c), but for a winter month (February). During summer, the negative trend in total ice-ocean stress dampens the positive trend in atmosphere-ocean stress (a). However, during winter, the reduction in total internal stress (red line, Fig. d) outweighs the contribution of changing atmosphere-ice stress.

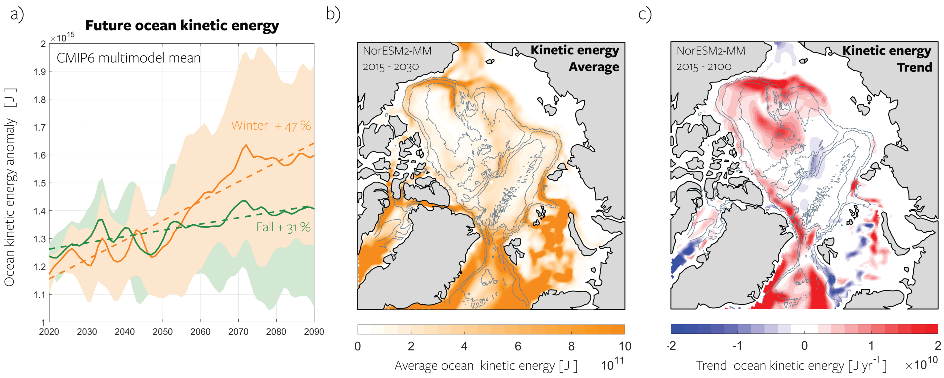


**Supplementary Fig. 3** Impact of changing surface stress on ocean kinetic energy. (a) Multimodel mean Arctic Ocean total kinetic energy integrated down to 100 m (anomalies relative to 2015–2030 model mean) projected by the CMIP6 models under a high-emission scenario for fall (green) and winter (orange). Envelopes indicate the model spread as determined by one standard deviation, and values indicated in the panel represent the seasonal increase from 2015 to 2100. Time series represent a basin-wide average and have been smoothed using a low-pass filter with a five-year cutoff frequency. Dashed lines represent the linear trend until 2100 and individual model trends are shown in Supplementary Fig. 1. (b) Spatial representation of the annual mean total kinetic energy integrated down to 100 m in NorESM2-MM. (c) Linear trend in annual mean total kinetic energy integrated down to 100 m in NorESM2-MM. Light grey contour lines show the 500 m and 2000 m isobaths.

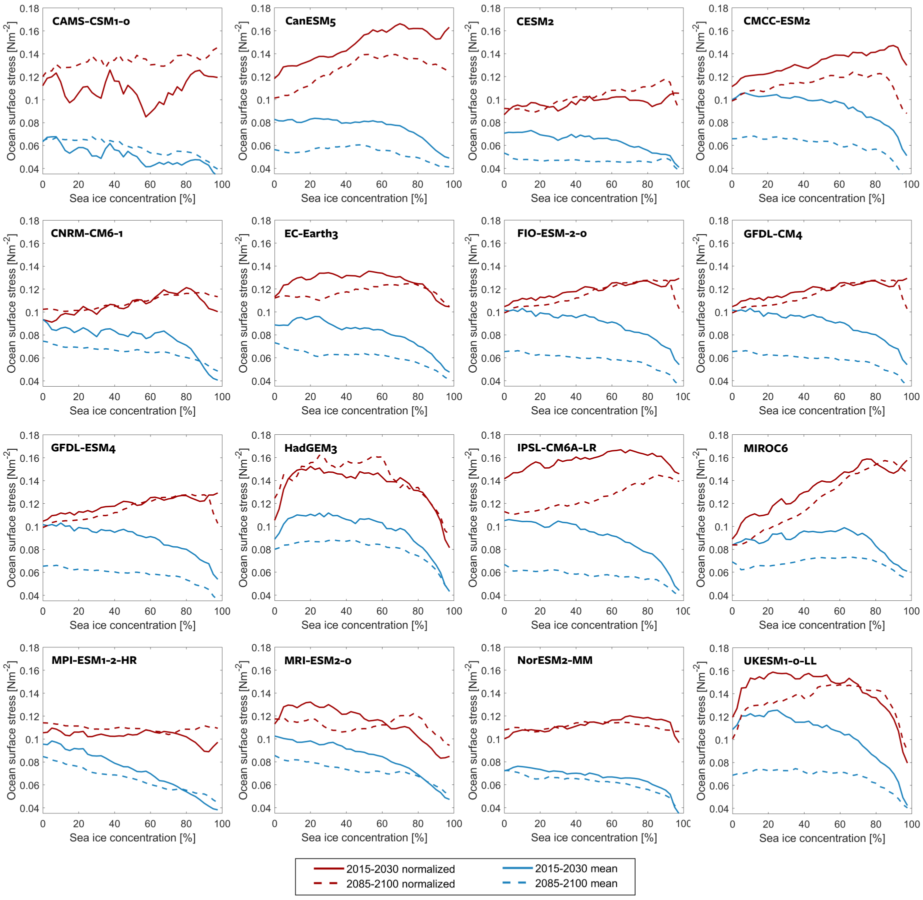


**Supplementary Fig. 4** Ocean surface stress as a function of sea ice concentration for all CMIP6 models. Sea ice concentration over the entire Arctic Ocean from monthly model output was binned using a bin width of 2%. For each bin, the mean ocean stresses were calculated—weighted by grid cell area for each year of the periods 2015–2030 and 2085–2100. Blue lines indicate the actual binned data whereas red lines show the binned data normalized by the square of the wind speed. Note the first bin on the left does not include open water (fully open water can have higher stress).
